# Encapsulation of InP/ZnS Quantum Dots into MOF-5 Matrices for Solid-State Luminescence: Ship in the Bottle and Bottle around the Ship Methodologies

**DOI:** 10.3390/ma17133155

**Published:** 2024-06-27

**Authors:** Alexis Tran, Rodolphe Valleix, François Réveret, Lawrence Frezet, Federico Cisnetti, Damien Boyer

**Affiliations:** Université Clermont Auvergne, Clermont Auvergne INP, CNRS, ICCF, F-63000 Clermont-Ferrand, France; alexistran27@gmail.com (A.T.); rodolphe.valleix@ens-lyon.fr (R.V.); francois.reveret@uca.fr (F.R.); lawrence.frezet@uca.fr (L.F.)

**Keywords:** MOF, QDs, InP, phosphor, luminescence

## Abstract

The utilization of InP-based quantum dots (QDs) as alternative luminescent nanoparticles to cadmium-based QDs is actively pursued. However, leveraging their luminescence for solid-state applications presents challenges due to the sensitivity of InP QDs to oxidation and aggregation-caused quenching. Hence, an appealing strategy is to protect and disperse InP QDs within hybrid materials. Metal–organic frameworks (MOFs) offer a promising solution as readily available crystalline porous materials. Among these, MOF-5 (composed of {Zn_4_O}^6+^ nodes and terephthalate struts) can be synthesized under mild conditions (at room temperature and basic pH), making it compatible with InP QDs. In the present work, luminescent InP/ZnS QDs are successfully incorporated within MOF-5 by two distinct methods. In the bottle around the ship (BAS) approach, the MOF was synthesized around the QDs. Alternatively, in the ship in the bottle (SIB) strategy, the QDs were embedded via capillarity into a specially engineered, more porous variant of MOF-5. Comparative analysis of the BAS and SIB approaches, evaluating factors such as operational simplicity, photoluminescence properties, and the resistance of the final materials to leaching were carried out. This comparative study provides insights into the efficacy of these strategies for the integration of InP/ZnS QDs within MOF-5 for potential solid-state applications in materials chemistry.

## 1. Introduction

Quantum dots (QDs) are a well-known class of emerging materials which find applications in many forefront fields such as biological imaging [1], lasers [2], displays [3], and solid-state lighting [4]. These semi-conducting nanoparticles feature electron confinement that provides exceptional optical properties, such as high photoluminescent quantum yields (PLQY), size-dependent emission tuning, narrow emissions, and a wide absorption range [5]. The most used QDs contain cadmium, but their use is increasingly restricted due to toxicity concerns (e.g., REACH regulations in Europe). Indium phosphide (InP) QDs are the most widely studied alternative [6,7,8,9]. For a long time, the luminescence properties of InP QDs were considered far less effective than those of their cadmium-based counterparts in terms of PLQY, particularly under prolonged photonic stress [10]. Furthermore, their susceptibility to rapid oxidation in ambient air severely constrains their applicability [11,12]. Recent advances have finally produced InP QDs that compare well with their Cd-based counterparts [9]. Additionally, similar to other quantum dots (QDs) [13], InP QDs experience the aggregation-caused quenching (ACQ) effect, restricting their utilization in some solid-state applications. To stabilize and avoid aggregation of InP QDs, the use of a protective inorganic matrix or hybrid host such as metal–organic frameworks (MOFs) seems an attractive strategy. MOFs are a family of compounds consisting of an ordered assembly of metal ions (or metallic clusters) and organic ligands (linkers), resulting in the formation of a single-, two- or three-dimensional network [14]. MOFs are therefore considered to be large-porosity materials with high specific surface areas, ideal as host matrices for nanoparticle encapsulation. Their potential applications can span from gas storage to sensors and catalysis [15,16,17]. Several studies have already demonstrated the effectiveness of encapsulating QDs in MOF-type matrices for solid state dispersion or for enhanced protection against external stresses. Either in “onto” or “into” modes, MOF@QD materials find applications, as reviewed for energy, photocatalysis, or light-harvesting system [18,19,20]. However, only one example is reported for InP with a ZIF-8 MOF (in the context of sensing) [21], highlighting the rarity of InP/MOF composites as compared to other QD/MOF materials (see, for instance, recent reviews [22,23]) which we relate to the relative fragility of indium phosphide as compared to other QDs (e.g., cadmium-based). This may be ascribed to an incompatibility of InP QDs with the MOF synthetic procedure (usually solvothermal) or with the conditions allowing its incorporation in preformed MOFs.

Overall, as far as luminescent materials are concerned, examples of MOF/QD composites (with any type of QDs) remain scarce [19,24]. However, on the one hand, it has been possible to embed InP/ZnS in other matrices such as silica shells [25] or layered double hydroxides (LDHs) [26], and, on the other hand, luminescent MOFs with cadmium-based QDs are still actively considered [27,28]. Thus, encapsulating InP QDs could be of high interest as compared to the state of the art in luminescent MOFs [29,30].

To achieve the encapsulation of InP/ZnS QDs, we selected MOF-5. This archetypical MOF [31], composed of {Zn_4_O}^6+^ clusters and terephthalate linkers [32,33], was chosen for its low cost and ease of synthesis, including by a well-known short (2.5 h) room-temperature protocol in basic conditions [34]. However, the pore size of MOF-5 (<2 nm) does not allow direct encapsulation of QDs (3–10 nm) [35]. We therefore need to find alternatives for integrating these nanoparticles into structures. 

In the present study, we investigate the encapsulation of InP/ZnS quantum dots (QDs) employing two distinct methodologies: the “bottle around the ship” (BAS) and the “ship in the bottle” (SIB) approaches [19,36]. In the BAS method, we performed the synthesis of the metal–organic framework (MOF) around the quantum dots. This involves an initial ligand exchange process to disperse the QDs within the solvent used for the MOF synthesis, namely *N*,*N*-dimethylformamide (DMF). We chose a room-temperature protocol [34], considering its compatibility with delicate nanoparticles like InP also due to the mitigation of strong acidity commonly associated with traditional solvothermal procedures, achieved by employing triethylamine (Et_3_N) as a mild organic base. Alternatively, in the SIB approach, we modified the original MOF-5 synthesis by introducing cetyltrimethylammonium bromide (CTAB). CTAB has been previously identified as a template agent for creating mesoporous MOF-5 structures under solvothermal conditions [37]. This modification enabled the incorporation of QDs into the MOF structure through capillarity. 

These two distinct approaches allow us to explore and compare different strategies for the encapsulation of QDs within MOF frameworks, offering insights into their effectiveness and potential applications. For both strategies, electron microscopy, X-ray diffraction (XRD) and nitrogen physisorption were employed to characterize the obtained materials. The quantities of QDs incorporated into the different matrices were determined by UV-vis spectroscopy and ICP-OES. Finally, the solid-state luminescence properties of the newly prepared MOF@QD materials are presented. 

## 2. Materials and Methods

### 2.1. InP/ZnS QDs

The QDs synthesis and characterization was carried out following an established procedure [26]. After synthesis, the QDs were suspended in chloroform and stored in a fridge with a concentration of 60 g.L^−1^. These suspensions remain stable for months. Structural and optical characterizations are described in Figures S1 and S2 (Supplementary Materials).

### 2.2. OLAm-Capped InP/ZnS with 6-Mercaptohexan-1-ol (MCH)

The ligand exchange of oleylamine-capped InP/ZnS QDs was performed following a modification of an existing method (Supplementary Materials) [38]. The resulting red solid was then suspended in an adjusted volume of DMF to obtain a 60 g.L^−1^ suspension of QDs before being stored in a fridge. Suspensions remain stable for several months. Structural and optical characterizations are described in Figures S3 and S4. 

### 2.3. Synthesis of MOF@QD

The MOF-5 material was synthesized at room temperature using a protocol described by Tranchemontagne et al [34] (Supplementary Materials). For the preparation of MOF@QD samples, the same synthesis was performed, but in each reaction mixture, a certain volume of the 60 g.L^−1^ DMF suspension of MCH-InP/ZnS QDs was added (0.1 to 0.3 mL corresponding to 6 to 18 mg of QDs). Other volumes were adjusted to conserve the total volume. The material purification method was the same as for pristine MOF-5. 

### 2.4. Synthesis of Mesoporous MOF-5/CTAB

Mesoporous MOF-5 was prepared using CTAB as template agent, adapting the synthesis of MOF-5 at room temperature as described in the Supplementary Materials. In a typical run, terephthalic acid (0.085 g, 0.5 mmol, 1 eq.), triethylamine (0.135 mL, 1 mmol, 2 eq.) and CTAB (ranging from 18 mg to 54 mg, corresponding to 0.1–0.3 eq.) were dissolved in 5 mL of DMF. Separately, zinc acetate dihydrate (0.285 g, 1.3 mmol, 2.6 eq.) was dissolved in 5 mL of DMF. The zinc salt solution was added to the organic solution, which was magnetically stirred at 700 rpm for 2.5 h. The precipitate was collected by centrifugation (10,000 rpm, 15 min). Then, it was subjected to washing with 1 × 15 mL of DMF to remove the excess of zinc salt, and 2 × 15 mL chloroform to remove the residual organic ligands while recovering each time the solid by centrifugation (same conditions as above). The powder was dried at 60 °C overnight.

### 2.5. Preparation of MOF@QD/x CTAB Samples

Hybrid materials were prepared by using 0.1 g of MOF-5/x CTAB powder, to which 0.3 mL of a suspension of InP/ZnS QDs (60 g.L^−1^ in chloroform, 18 mg) and 5 mL of chloroform were added. After a quick initial swirling to mix components, the vial was heated without stirring at 60 °C using a hot sand bath until total evaporation of the solvent. The final material was suspended in chloroform and then washed 3× with 10 mL of chloroform. The precipitate was collected by centrifugation (11,000 rpm/10 min) between the different washes. The final material was then dried at 60 °C overnight before analysis. 

#### Characterizations

X-ray diffraction experiments were performed with an X’Pert Pro diffractometer (PANalytical, Almelo, The Netherlands) with a Cu-Kα radiation (λ = 0.15418 nm) over 2θ angles ranges from 5 to 50°. Scanning electron microscopy was performed on a Jeol 6060-low vacuum apparatus (JEOL, Tokyo, Japan). The samples were prepared by deposition on a carbon tape and gold sputtering metallization. Nitrogen physisorption was performed on a Micromeritics 3-Flex (Micromeritics, Norcross, GA, USA) in the Chemical institute of Rennes (Rennes, France). The different samples were degassed at 200 °C for 24 h before analysis. All calculations: Brunauer, Emmet, and Teller (BET) and Barret, Joyner, and Halenda (BJH) are performed using 3Flex software V6.03 developed by Micromeritics. Photoluminescence quantum yield measurements (PLQY) and fluorescence spectra (including EEM) were recorded with an integrating sphere measurement system from Hamamatsu photonics (C9920-02G; Shizuoka, Japan). The system is composed of a 150 W Xenon lamp and integrating sphere coated with Spectralon and a CCD camera for the detection. The internal photoluminescence quantum yield PLQY_int_ and the absorption coefficient (Abs) defined by Formulae (1) and (2) were obtained directly from the measurements made in the integrating sphere. The external photoluminescence quantum yield PLQY_ext_ was calculated from the product of PLQY_int_ and Abs, and corresponds to the number of emitted photons over the number of incident photons (Equations (1)–(3)).

PLQY_int_ = n. emitted photons/n. absorbed photons (1)

Abs = n. absorbed photons/n. incident photons(2)

PLQY_ext_ = PLQY_int_ × Abs = n. emitted photons/n. incident photons. (3)

Semi-quantitative emission spectra were recorded using a 375 nm picosecond laser diode (75 ps and 20 MHz for the pulse duration and the repetition rate) combined with a 300 mm focal length monochromator (FLS 980; Edinburgh Instruments Ltd., Livingston, UK) and a photomultiplier Hamamatsu R928P. UV assays and fluorescence spectra in solution were performed with a Duetta Fluorescence and Absorbance spectrometer (Horiba, Inc., Chicago, IL, USA). ICP-OES analyses for indium were conducted using an ICP-OES 5800 Agilent (Santa Clara, CA, USA) in an axial mode at 230.606 nm. The solid samples were first mineralized. 50.0 mg of powder were introduced into a hermetically sealed Teflon microwave reactor (MW5000 Anton Paar, Graz, Austria) with 2 mL HCl (37%) and 6 mL HNO_3_ (68%). The reactors were placed in an Anton Paar microwave oven and heated (10 °C/min ramp) to 230 °C for 30 min. The acidic solutions were then recovered and diluted in a 50 mL volumetric flask with deionized water prior to ICP analysis. Scanning transmission electron microscopy coupled with a high-angle annular dark-field detector (STEM-HAADF) was performed on QDs in suspension in CHCl_3_ using a FEI Titan Themis microscope (Thermo Fisher, Waltham, MA, USA) operating at 200 kV; QDs were dropped on a Lacey Carbon membrane. QDs were dried at room temperature before introducing them into the microscope.

## 3. Results and Discussion

### 3.1. Synthesis of QDs

**Figure 1:**
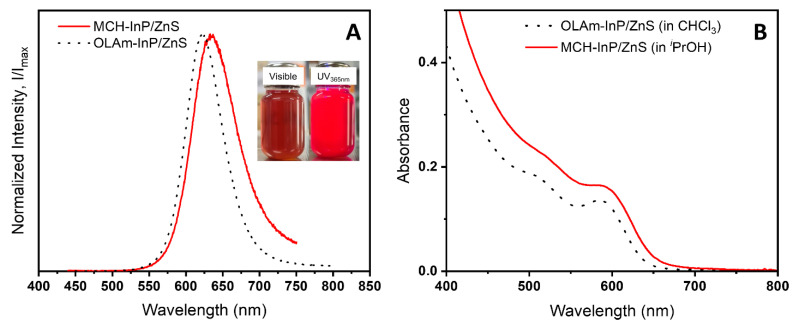
(**A**) Emission and (**B**) absorption spectra of InP/ZnS QDs before (dotted line) and after (red) ligand exchange with 6-mercaptohexan-1-ol. Inset in (**A**): photographs of MCH-InP/ZnS under visible and UV (λ_exc_ = 365 nm).

InP/ZnS QDs were synthesized following an established procedure [26]. They exhibit a bright red emission signal centered at 625 nm (Figure 1 and Figure S2), they present a photoluminescence quantum yield (PLQY) in solution of 50%. However, these nanoparticles experience aggregation-caused quenching (ACQ) and exhibit a solid-state PLQY_ext_ at 370 nm of 5.6% (Figure S2b). Then, in order to improve their compatibility with the synthesis medium of the MOF, we intended to perform a surface ligand replacement. In order to do this, the native oleylamine was replaced with 6-mercaptohexan-1ol (MCH). After several washing steps, the QDs were DMF-dispersible, thus compatible with an MOF synthesis. We will later refer to the QDs obtained through this procedure as MCH-QDs (or MCH-InP/ZnS). Only slight changes are noticed after the ligand exchange. A slight red shift of both the absorption and emission of the QDs was observed, but the width of the emission remains constant (Figure 1).

### 3.2. The Bottle around the Ship (BAS) Approach 

MOF-5 matrices have been synthesized in presence of MCH-InP/ZnS QDs. Over the course of the synthesis, no change in the color of the solution was noticed. Additionally, upon UV light excitation (λ_exc_ = 365 nm) no alteration was visible in the luminescence of the solution. At the end of the synthesis and after several washing steps, the obtained material remained red, highlighting the presence of the QDs in the MOF-5 structure (Figure S5). To quantify the amount of QDs incorporated in the hybrid materials, we performed a spectrophotometric analysis of MCH-InP/ZnS QDs still present in suspension in DMF after the MOF-5 synthesis. Briefly, the quantity of integrated (InP)_i_ is determined by calculating the difference between the quantity of MCH-QDs employed in the synthesis and the quantity of MCH-QDs still detected in the supernatant. The quantity of (InP)_i_ is calculated using the equations given in the Supplementary Materials from the absorbance at 413 nm. 

In all syntheses, the majority of QDs is incorporated into the structure (Table 1), with values close to 70% for all the samples. By considering the mass obtained for the different samples after synthesis, it is possible to give a value for incorporated InP in mmol per gram of material. These values can be confirmed by ICP-OES (Table 2).

**Figure 2:**
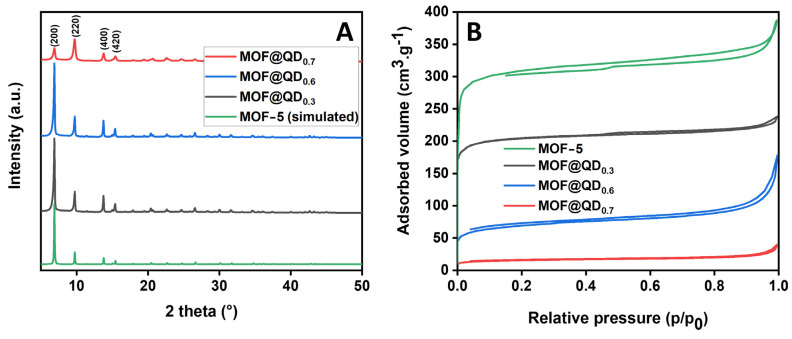
(**A**) Powder XRD pattern and (**B**) adsorption and desorption isotherms of MOF-5 [31] and synthesized MOF@QD_x_ (x = 0.3, 0.6, 0.7).

Then, we studied the impact of the amount of QDs on the structural and morphological properties of the samples. Despite the presence of InP/ZnS QDs in the sample, the powder XRD patterns of the synthesized samples are similar to those reported in the literature. The presence of QDs in MOF-5 matrices cannot be highlighted with this technique because the concentration of QDs incorporated is too low. The (200), (220), (400), and (420) main diffraction peaks of MOF-5 (Figure 2A) are present at 2θ = 6.8°, 9.7°, 13.7°, and 15.4°, respectively, suggesting a microcrystalline powder [32]. For the MOF@QD_0.7_, we noticed an inversion of the intensity ratio between the diffraction peak at 2θ = 6.8° and 9.7°, which may arise from the presence of lattice interpenetration and zinc-based species within the MOF pores [39].

To identify the presence of particles in the pores of MOF-5, we measured the specific surface area by nitrogen physisorption after the insertion of QDs in the structure. The various isotherms are of type I according to the IUPAC classification, evidencing the microporous nature of the various samples analyzed. By comparing the isotherms of MOF@QD_x_ with a classic MOF-5 synthesized in the same conditions, we observed a decrease of the specific area with increasing QDs loading rate (Figure 2B). 

Logically, as the loading of QDs increases, the specific surface area of the various samples decreases, resulting from the increasing congestion of the porosity. In addition to the fact that the luminescence of MOF@QD_x_ is conserved after several washings, these measurements seem consistent with the encapsulation of QDs within the MOF-5 matrix (Table 3).

The near-complete disappearance of the initial specific surface area of MOF-5 in the sample prepared with the highest initial quantity of QDs strongly indicates that this loading approaches its maximum capacity. Interestingly, our observations reveal that despite the increased QDs quantity during synthesis, the pore size (calculated using the BJH method) remained constant and consistently smaller than the size of QDs. This finding suggests that if the QDs are encapsulated, it does not occur through direct insertion into the pre-existing MOF porosity; rather, it happens through the formation of the matrix around the QDs during the synthesis process.

**Figure 3:**
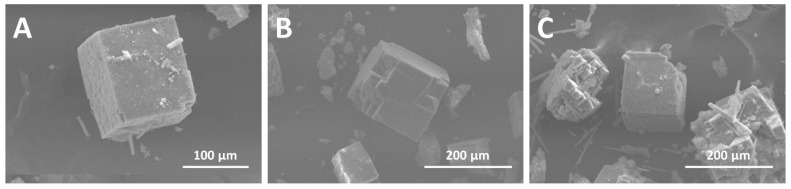
SEM images of (**A**) MOF@QD_0.3_, (**B**) MOF@QD_0.6_, and (**C**) MOF@QD_0.7_.

SEM images reveal the presence of cubic crystals, with an average size of 100–150 µm, corresponding to the microscopic organization expected for MOF-5 (Figure 3). However, the greater the initial quantity of QDs in the synthesis, the more the structure exhibits apparent defects. Cracks and even different microstructures were observed, particularly in the MOF@QD_0.7_ sample: the QDs create large defects in the structure, causing irregularities in the crystal appearance.

**Figure 4:**
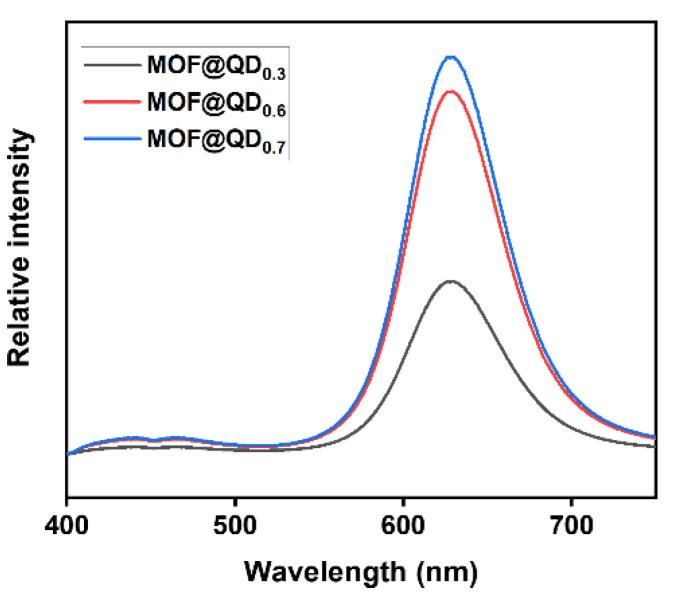
Emission spectra of solid-state samples of MOF@QD_x_ synthesized by the BAS approach (λ_exc_ = 375 nm).

The optical properties of the above obtained materials were then investigated (Figure 4). The maximum emission wavelength of those samples is at 628 nm (a slight hypsochromic shift compared with 634 nm for QDs after ligand exchange). The two samples with the highest QDs loading (for x = 0.55 and 0.69) present emission intensities that are close. However, they display twice the emission intensity of the MOF@QD_0.3_. The emission of the samples tends towards a maximum intensity as a function of the loading ratio. Doubling the amount of QDs in solution during the integration step does not double the maximum emission intensity. In MOF@QD_0.7_, reabsorption or aggregation phenomena (several QDs stacked within a pore) may occur, which could explain the lower intensity.

The PLQY of the different samples were compared (Table 4) to verify the hypotheses set out above. The PLQYint decreases with increasing QDs loading, while the absorption coefficient increases. However, the slight drop in PLQYint is offset by the increase in absorption coefficient, so that the sample with the best PLQYext is MOF@QD0.7 (consistent with the spectra, Figure S6).

Leaching tests were performed on these samples, leaving the powders in chloroform for several hours. No decrease in intensity was observed over time, showing once again that the particles are well confined inside the MOF (Figure S7).

### 3.3. The Ship in the Bottle (SIB) Approach

For the SIB approach, as the porosity of MOF-5 does not allow the direct encapsulation of QDs by capillarity—the average pore size being only 4 nm—we sought to prepare mesoporous MOF 5. For the sake of simplicity, we first decided to check whether the modification of the solvothermal preparation of MOF-5 by addition of CTAB was also transposable to our experimental conditions (i.e., room temperature and use of Et_3_N). The samples are designated as MOF-5/x CTAB, x being the molar ratio of CTAB used during the synthesis. 

**Figure 5:**
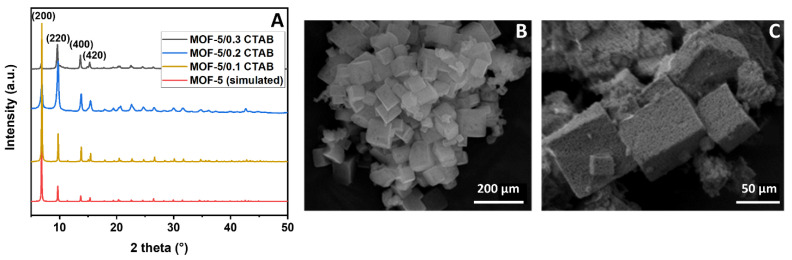
(**A**) Powder XRD patterns of simulated MOF-5 [31] and synthesized MOF-5/x CTAB (x = 0.1, 0.2, 0.3). SEM images of (**B**) MOF-5 and (**C**) MOF5/0.3 CTAB sample.

The XRD patterns recorded from MOF-5/x CTAB samples (Figure 5A) show diffraction peaks corresponding to the (200), (220), (400), and (420) reticular planes at 2θ = 6.8°, 9.7°, 13.7°, and 15.4°, respectively. While the sample synthesized with the least amount of CTAB exhibits a diffractogram similar to the simulated one, as the quantity of CTAB in the synthesis increases, the relative intensities of diffraction peaks of the (200) and (220) become inverted. However, despite this difference, the diffractograms still correspond to the expected structure for the MOF-5. 

**Figure 6:**
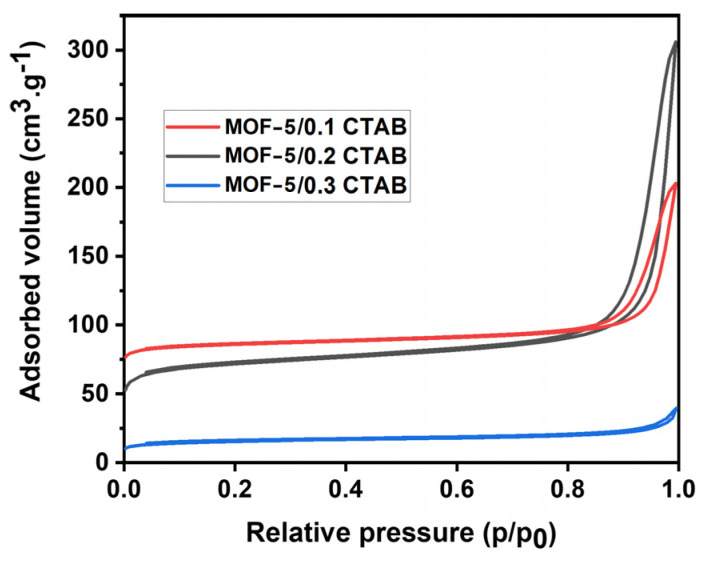
Adsorption and desorption isotherms of MOF-5/x CTAB (x = 0.1, 0.2, 0.3).

SEM image (Figure 5C) recorded from the MOF-5/0.3 CTAB displays a cubic morphology typical for MOF-5. However, the presence of CTAB in the synthesis seems to induce the formation of a new porosity. Indeed, asperities on the surface are undoubtedly visible on the images. This is in strong contrast with the smooth surface obtained in the case of unmodified MOF-5 (Figure 5B). Since the pore size is not accessible by direct SEM observation, MOF-5/x CTAB materials were analyzed by nitrogen physisorption to determine the new specific areas and porosities (Figure 6).

According to IUPAC classification, the isotherms correspond to type I. The higher the amount of CTAB in the synthesis, the lower the specific surface area of the MOF. This means that either the porosity becomes clogged, or the pore size increases, thus reducing the level of microporosity within the structure. The drop-off at desorption level indicated the presence of mesoporosity (Table 5). 

The specific surface areas measured are all much lower than the 940 m^2^·g^−1^ obtained with the MOF-5 (Table 3). Increasing the CTAB content decreases the specific surface areas of the MOF-5 and increases the pore opening values that are all larger than the size of the QDs (from 20 to 48 nm vs. 3–10 for typical QDs). These pore sizes are consistent with those reported by Ren et al. in solvothermal conditions 35. The modification of the MOF-5 synthesis involving the addition of CTAB made it possible to considerably increase pore size for direct encapsulation of nanoparticles without altering the structural properties of MOF-5. 

In each of these synthesized mesoporous matrices, 18 mg of MCH-InP/ZnS QDs are incorporated per 100 mg of MOF-5, which is the amount used previously for the BAS technique. After inserting QDs into matrices, samples are designated as MOF@QDs/x CTAB with x the molar ratio of CTAB used during the MOF-5 synthesis. The final powder obtained after insertion of QDs by this SIB protocol has a much more intense red color than that prepared by the BAS approach. Under UV excitation (λ_exc_ = 365 nm), the luminescence of these powders results in a strong red emission (Figure S8). Washing steps did not apparently diminish the luminescence of the final hybrid material, showing that the QDs were not simply deposited on the MOF surface but were well-incorporated within the structure. In stark contrast, the attempted insertion of QDs into MOF-5 prepared without CTAB failed as no red color nor luminescence was observable after the various washing steps. These initial indications underline the importance of CTAB in the preparation of MOFs, enabling larger porosity sizes to be obtained for the incorporation of InP/ZnS QDs. The different remaining solutions after the washing steps showed no luminescence. The amount of QDs incorporated was therefore the same for each sample and equal to the initial amount of QDs used. The amount of QDs embedded has been confirmed by ICP-OES measurements (Table 6).

**Figure 7:**
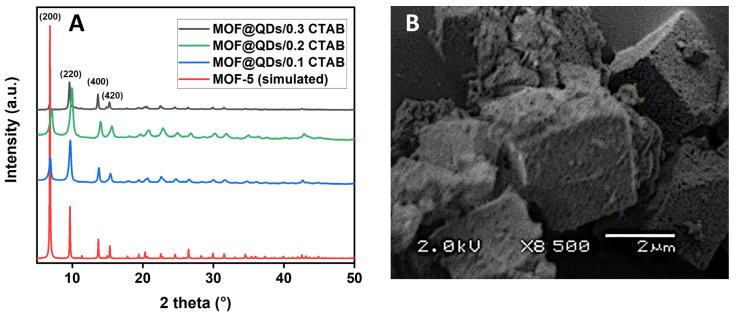
(**A**) Powder XRD patterns of simulated MOF-5 [31] and synthesized MOF@QDs/x CTAB (x = 0.1, 0.2, 0.3). (**B**) SEM image of MOF@QDs/0.3 CTAB sample.

The recorded diffractograms (Figure 7A) of the different MOF@QDs/x CTAB samples show no noticeable difference compared with samples synthesized without QDs. There is simply a slight shift of the diffraction peaks for the MOF@QDs/0.3 CTAB sample towards the low angles (2θ = 2°), which is currently not explained. The SEM image (Figure 7B) reveals the same morphologies as those obtained without QDs. CTAB-caused MOF-5 porosity is still apparent. The incorporation of QDs into the MOF-5 structure does not appear to alter the latter. 

The recorded diffractograms (Figure 7A) of the different MOF@QDs/x CTAB samples show no noticeable difference compared with samples synthesized without QDs. There is simply a slight shift of the diffraction peaks for the MOF@QDs/0.3 CTAB sample towards the low angles (∆2θ = 2°), which is currently not explained. The SEM image (Figure 7B) reveals the same morphologies as those obtained without QDs. CTAB-caused MOF-5 porosity is still apparent. The incorporation of QDs into the MOF-5 structure does not appear to alter the latter.

**Figure 8:**
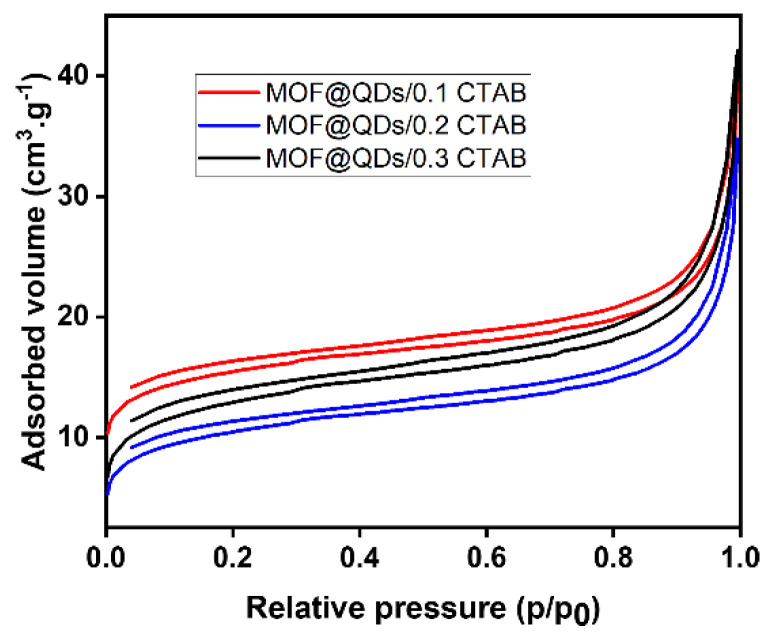
Adsorption and desorption isotherms of MOF@QDs/x CTAB (x = 0.1, 0.2, 0.3).

Then, nitrogen physisorption measurements were used to assess the evolution of the specific surface areas of samples after QD encapsulation (Figure 8). The isotherms still correspond to type I. However, specific surface area values decreased significantly after insertion of QDs (Table 7 vs. Table 5). These decreases may result from the insertion of QDs into the porosity of the materials, which completely obstruct the various pores. Measurement of the specific area allows us to conclude that the QDs are well-encapsulated within the MOF-5 crystals. 

**Figure 9:**
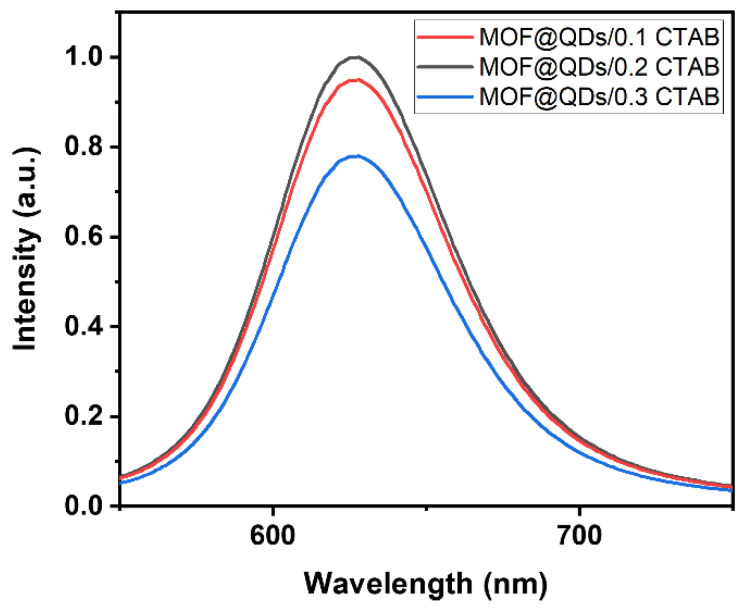
Emission spectra of MOF@QD/xCTAB samples (x = 0.1, 0.2, 0.3) recorded upon UV excitation (λ_exc_ = 375 nm).

Photoluminescence properties and PLQY were then measured on the various MOF@QDs/x CTAB samples obtained by the SIB approach. The maximum emission wavelength of all three samples is at 626 nm upon excitation at 375 nm (Figure 9). However, for the sample prepared with the highest amount of CTAB (i.e., 0.3 eq.), the emission intensity drops by ~20%. This result might be rationalized by the fact that the loss of porosity could result in aggregation of the QDs.

Measurements of PLQYs validate the hypotheses formulated by analysis of the emission spectra (Figure S9). Indeed, the quantity of QDs integrated seems similar in the three different samples, since the absorption coefficients of the different materials are comparable. The final material features high PLQY in the solid state, reaching 22% of PLQY_int_ for the MOF@QDs/0.1 CTAB and MOF@QDs/0.2 CTAB samples, whereas this value falls to 15% for the MOF@QDs/0.3 CTAB sample (Table 8). This decrease can be assigned to the well-known ACQ phenomenon between QDs InP/ZnS too close to each other within the structure.

Unlike materials synthesized by the BAS method, the porosity is large enough to accommodate QDs. In principle, this would also imply the possibility of a release of these QDs over time in the presence of solvent. This effect was quantified by measuring the evolution of the emission of the MOF@QD/x CTAB samples after several hours in chloroform. An amount of 0.100 g of MOF@QD/0.3 CTAB powder was placed in 10 mL of chloroform for several hours. For each time point, the powder was centrifugally rewashed and dried, then the luminescence spectrum was recorded (Figure S10). As expected, the emission intensity of the MOF@QDs/0.3 CTAB sample decreases over time in the presence of chloroform. However, after 10 h a limit (22% loss) seems to have been reached, with luminescence intensity almost unchanged up to 48 h. This study confirms that the SIB approach results in the majority of QDs being quite firmly embedded in the MOF-5 structure.

## 4. Conclusions

In the present work, we have investigated and compared two distinct methods aiming to incorporate InP/ZnS quantum dots (QDs) into the MOF-5 structure.

The *bottle around the ship* (BAS) method involves the synthesis of MOF-5 around the surface ligand exchanged InP/ZnS QDs. Despite the ease of MOF-5 synthesis (room temperature and mildly basic conditions), ligand exchange processes prior to and during synthesis cause a drastic reduction of the QDs’ PLQY. Thus, the resulting hybrid material exhibits red luminescence in the solid state with a maximum PLQY_ext_ of 7.2%.

On the other hand, the *ship in the bottle* (SIB) approach consists in the insertion of the QDs through capillarity within a pre-synthesized MOF possessing greater porosity. We modified the existing synthesis protocol used above by incorporating CTAB, serving as a structuring agent. This enabled the isolation of mesoporous MOF-5 at room temperature in basic conditions. The incorporation of QDs into the MOF-5 structure is accomplished simply through solvent evaporation. The final material obtained demonstrates superior luminescence properties in the solid state, yielding a maximum PLQY_ext_ of 18%. These materials outperform those materials prepared via the BAS method—both in terms of internal quantum yield and of higher QD loading.

While the SIB technique appears more promising for achieving bright luminescence, it is important to note that the materials prepared using the BAS method exhibit no leaching over time in the presence of solvent. The BAS approach allows the creation of solid-state luminescent materials with modest optical properties but larger specific surface areas, potentially advantageous in detection applications or when utilized with other types of QDs (*e.g.*, for catalysis). Despite a certain leaching phenomenon observed in the presence of solvent, the SIB approach facilitates the straightforward encapsulation of nanomaterials in matrices while retaining luminescence properties in the solid state. This comparative study provides insights into the trade-offs between optical performance, stability, and surface area, offering avenues for tailored applications of cadmium-free MOF@QD hybrids.

## Figures and Tables

**Table 1:** (InP)_i_ integration percentages in the various MOF@QD samples.

Mass of QDs	A_413 nm_	Initial InP (mmol)	Residual InP (mmol)	(InP)_i_ (mmol)	%Integration
6 mg	0.12	4.0 × 10^−2^	1.2 × 10^−2^	2.8 × 10^−2^	70
12 mg	0.21	8.0 × 10^−2^	2.2 × 10^−2^	5.8 × 10^−2^	73
18 mg	0.34	1.2 × 10^−1^	4.2 × 10^−2^	7.8 × 10^−2^	65

**Table 2:** Determination of the amount of (InP)_i_ in mmol/g of final material synthesized and designation for MOF@QD_x_ samples prepared by the BAS approach.

Mass of QDs	Mass Recovered (g)	Loading ^1^ (mmol/g) by UV-vis	Loading (mmol/g) by ICP-OES	Sample Designation
6 mg	0.113	0.28	0.30	MOF@QD_0.3_
12 mg	0.107	0.55	0.62	MOF@QD_0.6_
18 mg	0.101	0.69	0.75	MOF@QD_0.7_

^1^ mmol of (InP)_I_ per gram of final material.

**Table 3:** BET and BJH results for different MOF@QD_x_ samples synthesized by the BAS method.

Sample	S_BET_ (m^2^·g^−1^)	Pore Size (BJH) (nm)
MOF-5	940	4.5
MOF@QD_0.3_	655	4.4
MOF@QD_0.6_	234	4.3
MOF@QD_0.7_	51	4.8

**Table 4:** PLQYs measured for λ_exc_ = 365 nm on the different MOF@QD_x_ prepared by the BAS method.

Sample	PLQY_int, 365 nm_ (%)	Absorption Coefficient	PLQY_ext, 365 nm_ (%)
MOF@QD_0.3_	13	0.32	4.2
MOF@QD_0.6_	10	0.61	6.1
MOF@QD_0.7_	8	0.90	7.2

**Table 5:** BET and BJH analyses on MOF-5/x CTAB samples.

Sample	S_BET_ (m^2^·g^−1^)	Pore Size (BJH) (nm)
MOF-5/0.1 CTAB	329	20
MOF-5/0.2 CTAB	233	21
MOF-5/0.3 CTAB	50	48

**Table 6:** Determination of the amount of (InP)i in mmol/g of final material by ICP-OES (SIB approach).

Sample	Loading ^1^ (mmol/g) by ICP-OES
MOF@QDs/0.1 CTAB	1.14
MOF@QDs/0.2 CTAB	1.11
MOF@QDs/0.3CTAB	1.12

^1^ mmol of (InP)_I_ per gram of final material.

**Table 7:** Specific surface area (BET) and pore size (BJH) analyses on MOF-5/x CTAB samples.

Sample	S_BET_ after Insertion ^1^ (m^2^·g^−1^)
MOF-5/0.1 CTAB	52
MOF-5/0.2 CTAB	45
MOF-5/0.3 CTAB	27

^1^ insertion of 18 mg of QDs in each sample.

**Table 8:** PLQYs measured for λ_exc_ = 365 nm on the different MOF@QD/x CTAB prepared by the SIB method.

Sample	PLQY_int, 365 nm_ (%)	Absorption Coefficient	PLQY_ext, 365 nm_ (%)
MOF@QDs/0.1 CTAB	22	0.71	15.6
MOF@QDs/0.2 CTAB	22	0.82	18.0
MOF@QDs/0.3 CTAB	15	0.82	12.3

## Data Availability

The original contributions presented in the study are included in the article/Supplementary Materials, further inquiries can be directed to the corresponding authors.

## Supplementary Materials

The following supporting information can be downloaded at: https://www.mdpi.com/article/10.3390/ma17133155/s1, Method for the determination of the concentration of InP QDs, Supplementary Figures: X-ray diffraction, microscopy, spectroscopic characterizations, PLQY measurements, photographs of samples (PDF).
