# Supplementary material for: Encapsulation of InP/ZnS Quantum Dots into MOF-5 Matrices for Solid-State Luminescence: Ship in the Bottle and Bottle around the Ship Methodologies
Source: Materials (Basel). 2024 Jun 27;17(13):3155. doi: 10.3390/ma17133155 (PMC11242582; doi:10.3390/ma17133155)
Supplement: Supplementary file 1 [file materials-17-03155-s001.zip › materials-3069561-supplementary.pdf]

SUPPORTING INFORMATION for

Encapsulation of InP/ZnS quantum dots into MOF-5 matrices for solid state luminescence: *ship in the bottle* and *bottle around the ship* approaches.

Alexis Tran[a], Rodolphe Valleix[a], François Réveret[a], Lawrence Frezet[a], Federico Cisnetti[a], Damien Boyer[a]**

[a] Université Clermont Auvergne, Clermont Auvergne INP, CNRS, ICCF, F-63000 Clermont-Ferrand

1. *Determination of the concentration of InP QDs*

The absorption of a photon will generate electron-hole pair which allows to determine the gap following the equation (1):

$$\lambda = \frac{h.c}{E^{gap}} \tag{1}$$

With λ the wavelength (nm) at the excitonic peak.

The concentration of InP units (InP)$_i$, in chloroform or DMF and the concentration of nanocrystals (Nx) have been determined using the equation (2) and (3), based on previous studies[1]

$$[InP]_i = A.\frac{\ln(10)}{\mu_{i,th} l\, V_M} \tag{2}$$

$$[Nx] = \frac{[InP]_i}{N} \tag{3}$$

A being the absorbance at 413 nm, l the optical path length (1 cm), V_M the molecular volume of InP (3.10^{-5} $m^3.mol^{-1}$), $\mu_{i,th}$ the intrinsic absorption coefficient ($7.49.10^6$ m^{-1} for $CHCl_3$ and $7.35.10^6$ m^{-1} for the DMF) and N the number of InP units in the crystal, N is given by equation (4):

$$N = \frac{4\, L^3}{6\sqrt{2}\, a_0^3} \tag{4}$$

Where, L is the edge length determined by STEM or by calculation through the UV-Vis spectrum, and a_0 is the lattice constant of InP (0.5869 nm).

[1] S. Yu *et al.*, *Nat commun* ***9****, 4009 (2018);* M. D. Tessier *et al.*, *Chem. Mater.* 2015, 27, 13, 4893-4898

2. Experimental details

Materials

See Valleix et al[2] for starting materials and solvents used in InP/ZnS QD preparation. For surface ligand exchange 6-mercapto-hexan-1-ol (97%) was purchased from Sigma-Aldrich and used as received. For MOF preparation: terephthalic acid (>98%), triethylamine (>99,5%), cetyltrimethylammonium bromide (>98%) and zinc acetate dihydrate (>99%) were purchased from Sigma-Aldrich and used as received. Solvents for ligand exchange and MOF synthesis (at least synthesis grade) were purchased from various suppliers and were used as received. Pristine MOF-5 was prepared at room temperature following the procedure by Tranchemontagne *et al.*[3] (the synthesis is identical to the one of MOF-5/CTAB described in the manuscript except the omission of the CTAB additive).

Synthesis of InP/ZnS QDs capped with 6-mercaptohexan-1-ol (MCH)

InP/ZnS QDs were prepared as described previously.[2] Briefly, the synthesis implied the preparation of InP cores followed by the deposition of a ZnS shell. As oleylamine (OLAm) was used as the synthetic medium, the InP/ZnS QDs were initially obtained with OLAm surface ligands. The QD samples are initially obtained as suspensions in chloroform (see the cited reference for details and figures S1-S3 for characterization of as-synthesized QDs). The latter suspension (0.9 mL, 54 mg of QDs for a suspension of 60 $g.L^{-1}$) was placed in a three-necked round bottom flask under argon. A solution of a large excess of MCH (2 mL, 11 mmol) in 5 mL of DMF was added to the suspension of QDs. The mixture was heated to 120°C with stirring (1000 rpm) and left to react for 30 min. During this time, the QDs and DMF formed an apparently homogeneous suspension. The mixture was cooled to room temperature. The QDs were purified by two cycles of precipitation/redispersion by using centrifugation (11 000 rpm,

[2] R. Valleix *et al., Adv. Mater.*, 2021, **33**, 2103411.
[3] Tranchemontagne, D.J. *et al. Tetrahedron*, 2008, **64**, 8553.

15 min) with a 25 mL mixture of isopropanol/toluene (1/5 – v/v). 0.06 g of a red solid were obtained.

3. *Supplementary figures*

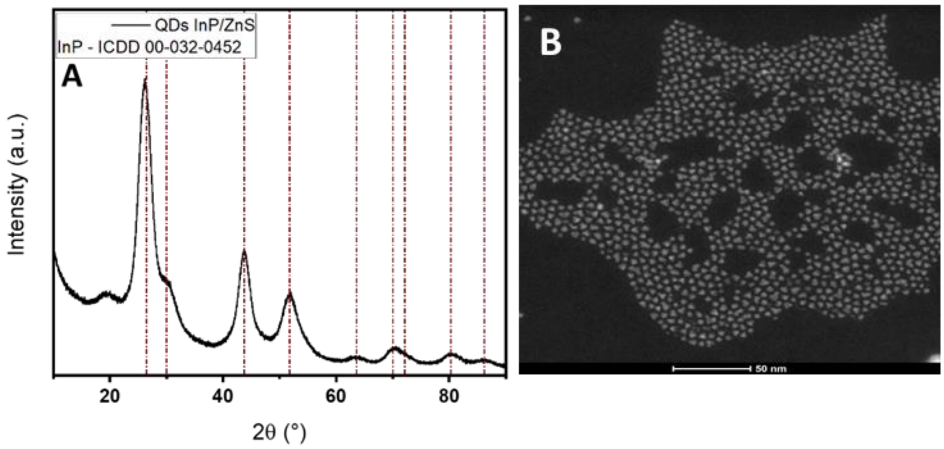

Fig. S1: (A) XRD diffraction pattern of InP/ZnS compared to the ICDD file n°00-032-0452. (B) STEM-HAADF image of InP/ZnS quantum dots.

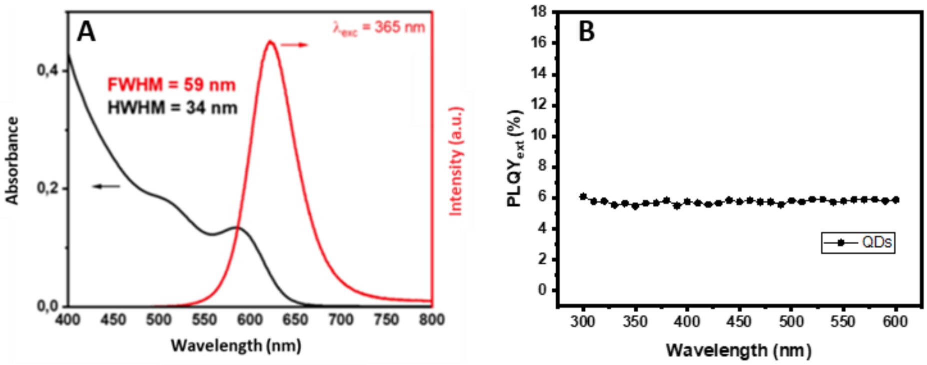

Fig. S2: (A) Optical characterizations of InP/ZnS QDs after synthesis. Emission spectrum (λ_{exc} = 365 nm) and absorption spectrum of InP/ZnS QDs recorded in chloroform. The maximum of $PLQY_{int}$ is 50% upon excitation at 365 nm. **(B)** $PLQY_{ext}$ of InP/ZnS QDs in solid state after evaporation of the chloroform.

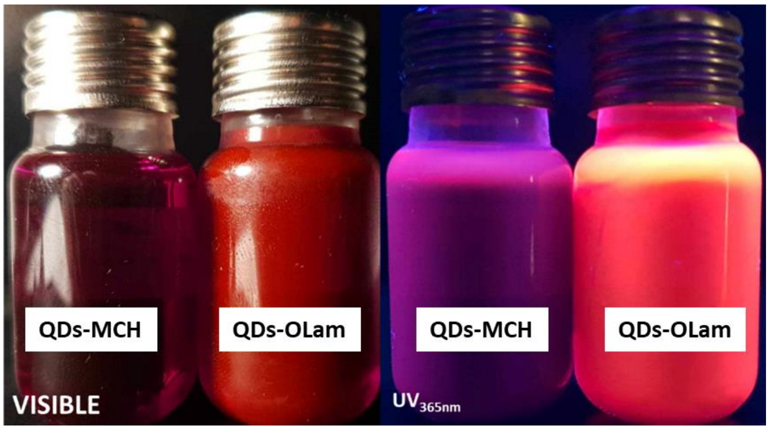

Fig. S3: Picture of OLAm-capped InP/ZnS QDs in chloroform and MCH-capped InP/ZnS QDs in DMF under visible light and UV_{365nm}.

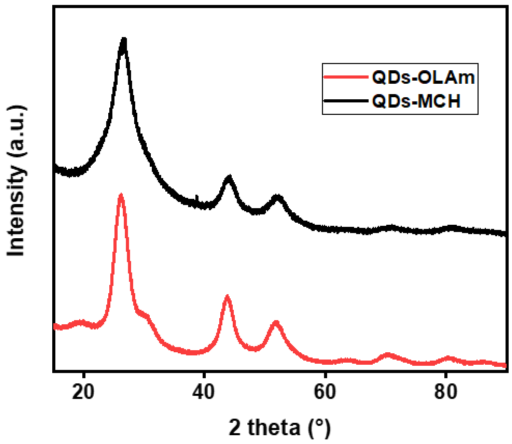

Fig. S4: XRD diffraction pattern of OLAm-capped and MCH-capped InP/ZnS QDs.

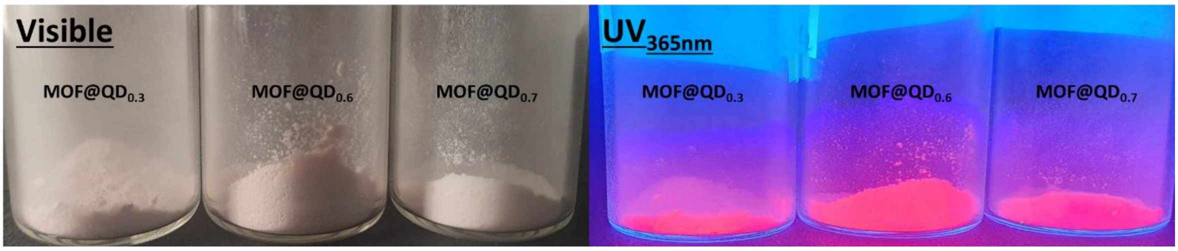

Fig. S5: Picture of MOF@QD samples prepared by the BAS approach, under visible light and UV_{365nm}.

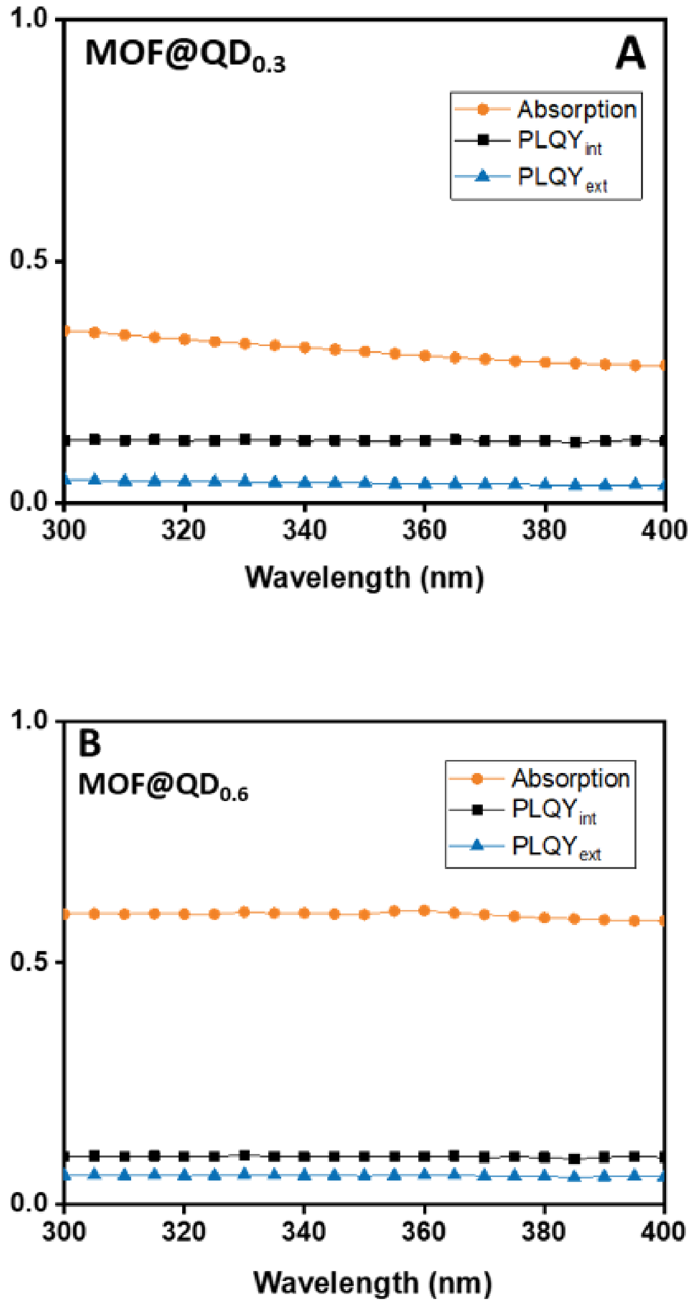

MOF@QD$_{0.3}$
A
Absorption
PLQY$_{int}$
PLQY$_{ext}$
1.0
0.5
0.0
300
320
340
360
380
400
Wavelength (nm)
B
MOF@QD$_{0.6}$
Absorption
PLQY$_{int}$
PLQY$_{ext}$
1.0
0.5
0.0
300
320
340
360
380
400
Wavelength (nm)

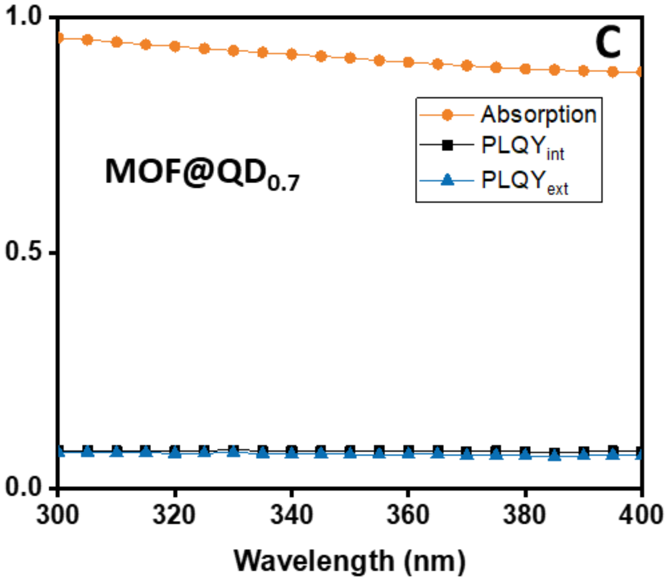

Fig. S6: PLQY$_{int}$, PLQY$_{ext}$ and absorption coefficient for the (A) MOF@QD$_{0.3}$, (B) MOF@QD$_{0.6}$ and (C) MOF@QD$_{0.7}$ samples prepared by the BAS at different wavelengths**.**

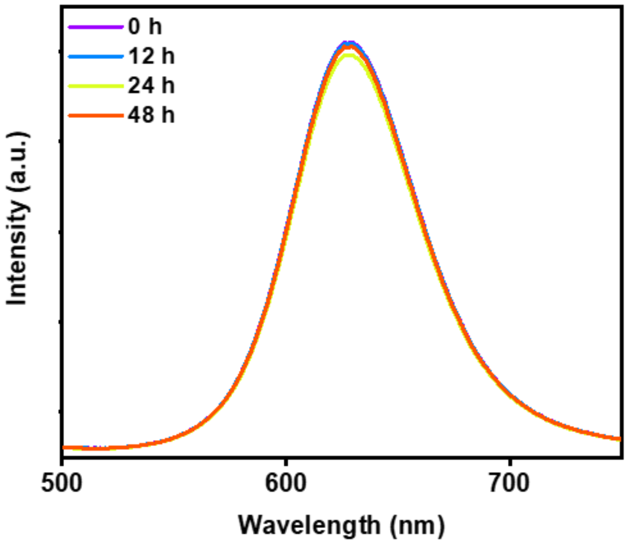

Fig. S7: Temporal evolution of the emission spectrum (λ_{exc} = 365 nm) of the MOF@QD$_{0.7}$ in chloroform over 48 hours.

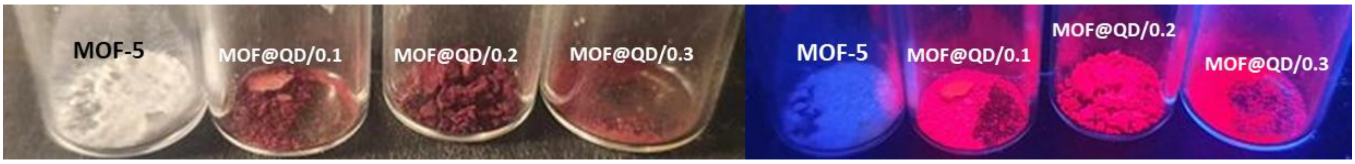

Fig. S8: Picture of the different MOF@QD/CTAB samples prepared by the SIB approach.

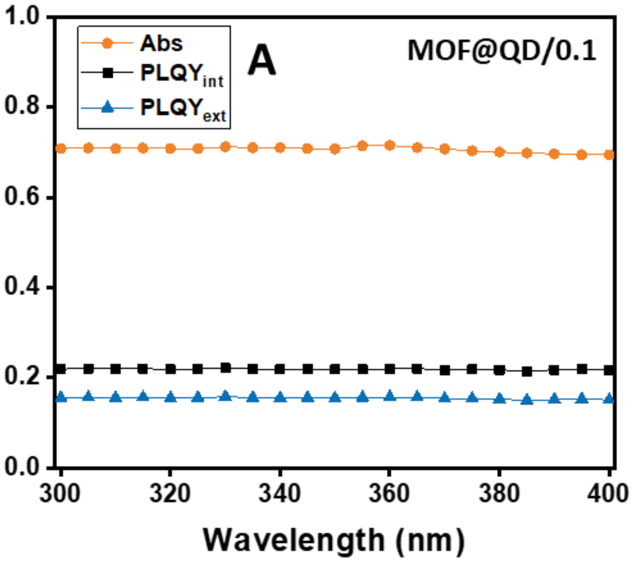

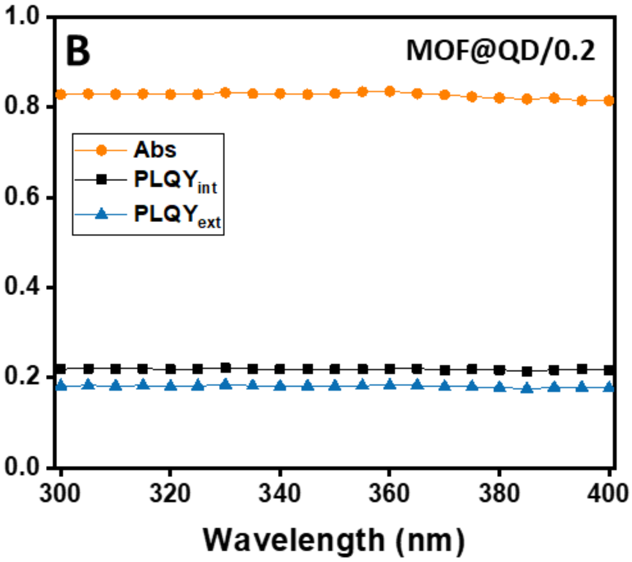

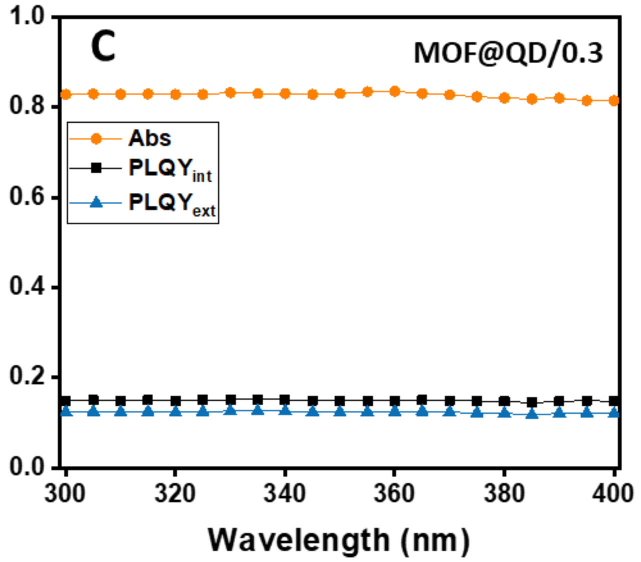

Fig. S9: $PLQY_{int}$, $PLQY_{ext}$ and absorption coefficient for the (A) MOF@QD/0.1 (B) MOF@QD/0.2 and (C) MOF@QD/0.3 samples prepared by the *ship in the bottle* at different wavelengths.

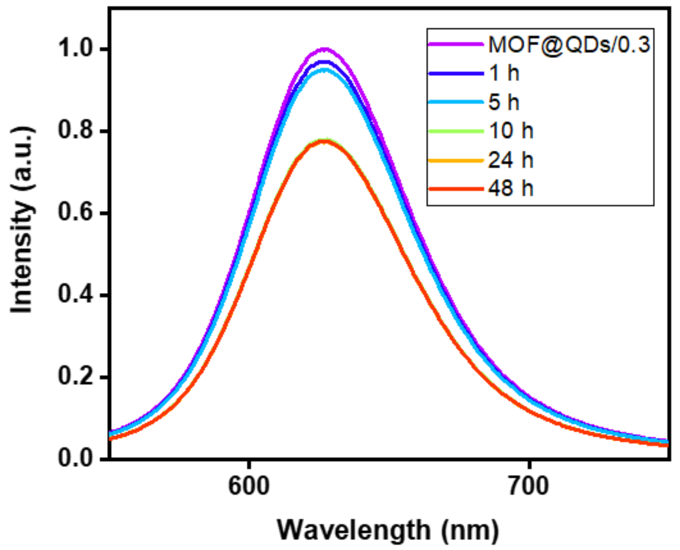

Fig. S10: Temporal evolution of the emission spectrum (λ_{exc} = 365 nm) of the MOF@QD/0.3 CTAB in chloroform over 48 hours.
